# The Inspection of Dairies1Read before the State Sanitary Association at the Laurel House, Lakewood, New Jersey, December 11, 1896.

**Published:** 1897-07

**Authors:** George W. McGuire

**Affiliations:** Dairy Commissioner of New Jersey


### THE INSPECTION OF DAIRIES.[1]

By George W. McGuire,
DAIRY COMMISSIONER OF NEW JERSEY.

In discussing this subject it is my desire to awaken an interest in the minds of the members of this Association in regard to a more perfect system of milk-inspection in our State. The milk-laws now on our statute-books were placed there with a view to remedy the loose and criminal practices that were made use of in the debasement of the milk-supply for the profit of the seller. These laws have been in operation for about fifteen years, and while the object of the framers was of a commercial rather than a sanitary nature, an incalculable amount of good has resulted from their enforcement—to the dealer, financially, in that he has been protected against the buying of diluted milk; to the consumer, in that the dealer is subjected to an official watchfulness; and it is the privilege of both to prove their suspicions by asking for an official inspection of the milk they are receiving.

The inspectors employed by the State have become so expert in handling milk that it is difficult for anyone to continue long in the sale of adulterated milk without detection. Generally speaking, there is little contention on the part of the defendant in cases of prosecution for violations of the milk-law; when milk is subjected to a chemical analysis and found to contain less than 12 per cent. total solids, the fact is invariably accepted by all experts that water or skim-milk has been added to it, and it is very seldom that a person detected in watering his milk is willing to stultify himself by contesting the prosecution.

Owing to the geographical location of our dairy-farms, most of which are so accessible to markets requiring a fresh milk-supply, a very small percentage of the milk of this State is made into butter.

According to recent statistics there are in New Jersey 200,347 milch-cows, the value of which is $6,887,930. By this it will be seen that the dairy industry of the State is of vast importance and steadily increasing, while the necessity for a more thorough and systematic inspection as to the health of the cattle and their product is growing in proportion, especially so when it is remembered that

[1] Read before the State Sanitary Association at the Laurel House, Lakewood, New Jersey, December 11, 1896.

a large percentage of the milk from these cattle passes through so many hands before it reaches the consumer.

New York has a daily consumption of about 700,000 quarts per day, Philadelphia about half that quantity. A very large share of the milk going into the city of New York is either produced in or passes through our State, and it becomes necessary for the inspector to know something of the quality of the milk which is being transported. And in this connection I do not hesitate to say that the milk produced in our State is equal if not superior to that from other States.

It is the duty of the milk-inspector to see that the local dealers in our cities and towns dispense milk containing 12 per cent. total solids, and for this purpose it is necessary for him to be frequently at the receiving stations and creameries where milk is received from the farmers, to see that the wholesale dealers do not dilute the milk with skim-milk or water, and finally to visit the farm or dairy where milk is produced and inspect the premises and the methods there in use.

It is said by bacteriologists that, in addition to the bacteria peculiar to milk, innumerable others dangerous to life and health come from the air, the water, and many other sources, both animal and vegetable, and that milk at all temperatures between the freezing-point and 160° is in a complete state of readiness to receive and grow them. Yet many farmers are not willing to accept these scientific facts which have been so unquestionably proven of late years, and scout the idea of any harm resulting from the use of milk, however carelessly it may have been handled.

It is far from my desire to give the impression that persons engaged in producing milk are needlessly untidy, and the product thus of an unwholesome character, but rather there is a legitimate basis of cleanliness and proper methods to which all dairymen should conform, and when this is appreciated and heeded by milk-producers it will be conducive of health to the public and economy to the dairymen. There are, however, persons engaged in producing milk in this State that by their habits, education, and surroundings are totally unfit to handle and sell this important article of food, and according to the legal advice we have we are powerless to compel better conditions or cause the abolishment of the business. In his regular inspection-tours the trained milk-inspector soon discovers these places, and in his efforts to improve on the practices in use he is met almost at the start with the knowledge that the law is inadequate to assist him, frequently he is defied by the owner, and the milk which should be so pure and nourishing, but which, through the ignorance and negligence of the producer, is so dangerously unwholesome, continues to be consumed by the public day after day, until an epidemic breaks out, and we wonder what has caused it.

During the past year the speaker, in company with the Secretary of the State Board of Health, visited two or three premises on which milk was produced by persons of this character, and witnessed an exhibition of filthy methods and surroundings which I am sure would startle my hearers and awaken them to renewed efforts in the direction of securing larger official supervision over the milk-supply. To illustrate, I will mention two cases which came under my notice officially during the past year, showing to what extent ignorance and filth abound in the management of one dairy and intelligence and tidiness in another. The following is a copy of the notes taken on the premises of an alleged dairyman on Penn Horn Creek, Hudson County, on December 26, 1895.

Copy of report, December 26, 1895: "Visited the premises of a dairy near Penn Horn Creek. This place is reeking with filthy air—the ground, the water, the buildings, and the cattle. The owner has sixteen head of milch-cows, which are kept in a stable about thirty feet long and six feet and six inches high. This party collects swill in New York and feeds it to his cows; he boils portions of the swill for the purpose of recovering the fat contained in it. Garbage in piles, in barrels, and strewn upon the ground, together with excreta from the cattle, render the premises revolting. The cattle stand deep in their droppings, and their bellies, sides, and tails are plastered thick with dung. Several of the cows were thin, and two appeared to be emaciated by disease, but, notwithstanding the condition under which they are kept, the remainder were plump. The cans are washed with water from a well on the premises, and a barrel is used to hold the water during the washing or rinsing of the cans. This contains garbage. The water pumped direct from the well had an offensive smell. This man is said to sell his milk in Hoboken."

The following inspection was made on September 20th, at a farm in Wantage Township, Sussex County:

"This herd consists of thirty-two cows of native breed, which were brought from the pasture to the barn in the evening to be milked. The barn was large and airy, and had ample floor-space for each cow. The cows had the appearance of being in a fine condition as to health, and the barn was noticeably clean, no trace of manure appeared at the time of the inspection. The milking was done by the owner and a hired man, and their methods were closely watched while they performed their work. The first thing they did after the cows were placed in their stalls was to thoroughly brush the bellies and udders; they then went to a small room where soap and water was provided and thoroughly washed their hands and faces; when each man had milked two cows he would again go to the washroom and wash his hands. This continued until the whole herd was milked. As soon as the milk was drawn from the cows it was taken to an adjoining room, which had been freshly whitewashed, and filtered through a clean cloth and metal strainer; it was then passed over a milk-cooler and aërated, and when the milk had been thus treated the temperature was 55°. The milk was then taken to a milk-house and the cans submerged into spring-water to within eight inches of the top and left there until the following morning, when it was taken to the station for shipment to New York City.

"The milk-cans were thoroughly inspected and found to be bright and absolutely clean. The owner of the dairy was highly complimented by the inspector on his treatment of his cows and their product, and he stated that he had no difficulty whatever in disposing of his milk; that he was never requested to withhold it in times of a surplus in the market, and never had any return on account of its souring or from any other reason."

It is at once apparent that the existence of such a place as I have described, located in Hudson County, is a daily menace to the life and health of every person who consumes any part of its product, while, on the other hand, if every dairy in our State were conducted on the lines adopted by the Sussex County dairyman, and equal care was observed by all who handle it until it reaches the consumer, not only would the public health be benefited, but the knowledge that we are consuming a clean, wholesome food would add to our pleasure and comfort.

Another source of danger to the milk-supply is in the handling of milk by persons in poor health with germs of disease about them. The Massachusetts Board of Health say, in their report just issued, that one of the most common means by which typhoid fever is spread among the population is the milk-supply. One of the men employed in the dairy may show the first symptoms of typhoid, and continue at his work for days before he is actually stricken down with the fever. In the meantime he constantly handles the pans, utensils, and even the milk which the public, not suspecting the danger, will eventually drink, and in this manner the germs of disease are communicated to the milk from the hands of the man sickening of typhoid, and the consumer takes into his system the germs of a violent disease, which may or may not have a disastrous effect, according to his physical condition.

Perhaps no country in the world excels the little kingdom of Denmark in its methods of so handling milk as to assure its wholesomeness. With a population of 2,000,000 of people, and an area about half that of the State of Maine, they have made a reputation for their dairy-products over all Europe, to which they annually export nearly 2,000,000 pounds of butter. As a people they have been instructed in the art of dairying by the Royal Agricultural Society, which in 1837 started a school for the purpose of providing dairy-instruction for farmers' sons and daughters, and in addition to this there is an agricultural school for dairy science at Copenhagen. The influence of this school has been felt over all the kingdom, and the credit is due these institutions for the marked success which Denmark enjoys as a dairy nation.

In the city of Copenhagen competition in the supply of fresh milk is very sharp, but I am told it takes the form of supplying a fine quality rather than selling it at reduced prices.

One large firm organized for the purpose of furnishing its patrons with absolutely pure milk adopted a very strict code of rules governing the handling of milk, to which every seller conformed if he desired to sell his products to the firm. The rules relate to the quality and quantity of the food of the animals in winter and summer, and the wholesomeness of the milk before and after calving. They also provide that the hair from the udder, the tails, and hindquarters be clipped before stabling in the fall, and properly regard cleanliness in the milking, cooling, and filtering, cleanliness of milk-vessels, drainage, water-supply, etc. This company employs a veterinarian to watch the health of the cows, and he spends his time in travelling from farm to farm for this purpose. They also employ a staff of trained dairy-maids, who travel from farm to farm to examine the surrounding conditions with reference to the cleanliness and care in milking. They watch the feeding, the cooling of the milk, and its preparation for shipment. When the milk is received in the cities an experienced dairy-woman stands by and tastes and smells the milk from each farm, noting whether it falls below the high standard that is required. It is then immediately filtered through a large enameled bowl with a capacity of about forty gallons. This bowl is so constructed that the milk is entered at the bottom and is discharged from pipes on the sides near the top. The milk is poured into a large reservoir somewhat higher than the filter, and thus attains pressure sufficient to force it through several successive layers of gravel and six thicknesses of cloth, thus assuring the customer that it is absolutely clean, pure, and wholesome.

It would be a good stroke of business enterprise if the milk-dealers in our State were to study the methods in use in Denmark, with a view to improving the quality of the milk they handle, and at the same time secure to themselves a larger and more substantial business, with greater profits.

One of the greatest difficulties in the way of procuring desirable milk is the sharp competition in the price among the dealers. This has a disastrous effect upon the producer, who seldom receives a reasonable profit for his product. This condition of affairs in trade influences dairymen to keep a grade of cows which will give a larger flow of milk, irrespective of its quality, and affords no incentive to put in operation the more superior methods of a well-conducted dairy. There are a number of dealers in our State, however, who pay the producer a liberal price for his product and insist upon receiving milk which has been treated in such a way as to insure its absolute wholesomeness. These men are successful dealers, and deserve the support of every sanitarian and the encouragement of every intelligent citizen.

In addition to our present laws we need legislation which will give power to the local board of health of every municipality in the State to cause an inspection to be made of all premises where milk is produced, vesting such authorities with power to enforce proper sanitary regulations and requiring them to keep a book of registry for the purpose of recording the facts connected with each inspection, which record shall be open to the inspection of the State Board of Health or State Dairy Commissioner, or submitted to them upon request. A provision should also be made in the law that if the local authorities refused or neglected to enforce the law it should be lawful for the State Board of Health or State Dairy Commissioner to prosecute the offenders. If a measure embracing the above features be enacted into the law, the effect will readily be seen. By securing a registry list of all persons engaged in the milk-business the State departments would not only be in possession of the most valuable data, but also a way opened by which a most systematic inspection could be operated in every county and the public insured a pure, wholesome milk-supply.

